# Do humour styles moderate the association between hopelessness and suicide ideation? A comparison of student and community samples

**DOI:** 10.1371/journal.pone.0295995

**Published:** 2023-12-18

**Authors:** Aaron C. Drake, Christopher R. Sears

**Affiliations:** Department of Psychology, University of Calgary, Calgary, Alberta, Canada; University of Pecs Medical School, HUNGARY

## Abstract

Research has found that humour styles can moderate the relationship between various facets of mental health and well-being. Most of these studies have used college student samples, however, and the generalizability of these findings has not been firmly established. This study examined how humour styles moderate the relationship between hopelessness and suicide ideation in both student and community samples. Community participants from the U.S. and Canada (*n* = 554) and student participants from a Canadian university (*n* = 208) completed several self-report measures including the Humor Styles Questionnaire. Analyses revealed differences in humour styles between the samples, as well as differences in humour styles between men and women. Regression analyses showed that self-defeating humour moderated the relationship between hopelessness and suicide ideation for student participants but not for community participants. Conversely, self-enhancing humour moderated the relationship between hopelessness and suicide ideation for community participants but not for student participants. These results suggest that high levels of self-defeating humour and self-enhancing humour may be uniquely maladaptive for these respective samples. These and other findings point to the necessity of recruiting diverse samples to better understand the beneficial and detrimental associations between humour styles and mental health. The potential to use measures of humour style as a tool to help identify at-risk individuals and to inform the development of intervention programs is discussed.

## Introduction

Humour has been considered beneficial to psychological well-being for centuries, a claim found in the Old Testament (“A merry heart doeth good like a medicine”) and in modern proverbs (e.g., “Laughter is the best medicine”). Humour has been purported to contribute to psychological well-being in many different ways [1]. Positive emotions such as mirth, happiness, and joy are part of the experience of humour and laughter, and are linked to lower levels of neurotransmitters and hormones involved in the stress response [2–4]. Using humour to change one’s perspective in stressful situations can help alleviate stress and anxiety, and doing so habitually may be an effective coping strategy [5–8]. Humour is also recognized to contribute to the creation and maintenance of healthy social relationships, which provide stress-reducing and health-enhancing benefits [9–12].

Contrary to popular belief, however, the psychological literature on the beneficial effects of humour is not extensive and is not unequivocal. In fact, initial research on the influence of humour on mental health did not reveal strong positive benefits [13–16]. Specifically, the relationships between various self-report measures of humour and common measures of mental health–such as psychological well-being, self-esteem, and mood–were weak or inconsistent, suggesting there are no substantial mental health-related benefits of humour [14]. Subsequently, Martin et al. [17] noted that researchers had not considered the possibility that there could be different forms of humour that have different influences on mental health. Whereas many studies treated humour as an all-encompassing construct, Martin et al. proposed that there are adaptive and maladaptive forms of humour, and if not distinguished, the benefits of adaptive forms of humour would be difficult to discern. Although several measures of positive (adaptive) forms of humour have been developed, such as an individual’s propensity to smile or laugh in certain situations (the Situational Humor Response Questionnaire [18]), and the use of humour to cope with stressful experiences (the Coping Humor Scale [8]), no self-report measure distinguished between positive and negative forms of humour prior to Martin et al.’s [17] study. The Humor Styles Questionnaire (HSQ) was developed to address this limitation.

The HSQ assesses four dimensions of humour: affiliative, self-enhancing, aggressive, and self-defeating [17]. Affiliative and self-enhancing humour are positive, adaptive humour styles posited to be beneficial to mental health. Affiliative humour is the use of humour to elicit connections with others in a manner that is welcoming and benevolent and conducive to social interactions. Self-enhancing humour is the use of humour to benefit oneself with no harm to others or one’s own image, and is primarily used as a coping mechanism to deal with stressful or negative events: one uses self-enhancing humour to make light of a situation and keep a positive outlook despite adversities. In contrast, aggressive humour and self-defeating humour are considered negative, maladaptive humour styles posited to be detrimental to mental health. Aggressive humour is used to enhance oneself at the expense of others without consideration of other people’s feelings. Self-defeating humour is the use of humour to diminish oneself and make jokes at one’s own expense, typically in an attempt to make oneself more relatable and acceptable to others. According to Martin et al. [17], these four humour styles also distinguish self-focused (i.e., self-enhancing and self-defeating) and other-focused (i.e., affiliative and aggressive) forms of humour. Thus, the HSQ attempts to capture both the form and focus of an individual’s humour style, and by doing so, has the potential to provide better insight into associations between humour styles and mental health.

### Humour styles and mental health

Several studies have examined associations between the HSQ and various facets of mental health and well-being. Martin et al. [17] found that both self-enhancing and affiliative humour positively correlated with self-esteem, psychological well-being, and social intimacy, and negatively correlated with anxiety and depressive symptoms. In contrast, self-defeating humour was found to positively correlate with anxiety and depressive symptoms and negatively correlate with self-esteem, psychological well-being, social intimacy, and social support satisfaction. Several other studies have reported similar associations between humour styles and these facets of mental health, as well as optimism, life satisfaction, shyness, and hopelessness [19–24]. Together these findings suggest that adaptive humour styles may protect against negative mental health outcomes, whereas maladaptive humour styles may make one more vulnerable.

A few studies have examined moderating effects of humour styles on the associations between various mental health measures. Olson et al. [25], for example, found that self-enhancing and affiliative humour moderated the strength of the association between rumination and feelings of dysphoria in a sample of undergraduate students. More specifically, students with high levels of trait rumination and high levels of self-enhancing and affiliative humour had lower levels of dysphoria, suggesting that these humour styles help to protect at-risk individuals from developing clinically significant depression. Tucker, Judah, et al. [26] found that both affiliative and self-defeating humour significantly moderated the relationship between social anxiety disorder (SAD) and major depressive disorder (MDD) in a college student sample, but in opposite ways: students with high SAD scores and high levels of affiliative humour had lower MDD scores, whereas students with high SAD scores and high levels of self-defeating humour had higher MDD scores. According to Tucker, Judah, et al., their results suggest that high levels of affiliative humour can reduce the risk that MDD will be coupled with SAD by reducing an individual’s likelihood of experiencing negative social interactions. On the other hand, for those with high levels of self-defeating humour, the relationship between SAD and MDD is exacerbated and could ultimately be more harmful.

### Humour styles and suicide ideation

While a better understanding of how humour styles relate to depression and anxiety is necessary, the relationship between humour styles and suicide ideation is an important but understudied concern. Suicide was the 17^th^ leading cause of death globally in 2019 [27], the 10th leading cause of death in the United States in 2019 [28], and the 9^th^ leading cause of death in Canada in 2016 [29]. Many of the aforementioned risk factors for mental health issues, such as depression, low self-esteem, rumination, and lack of social support, have been shown to be closely related to suicide ideation [30–35].

Few studies have examined how humour styles relate to suicide ideation. Tucker et al. [36] found that self-defeating humour was a significant mediator of the relationship between rumination and suicide ideation in a college student sample. That is, they found that the relationship between rumination scores (as measured by the Ruminative Responses Scale [37]) and suicide ideation was partially explained by a student’s use of self-defeating humour. According to Tucker et al. [36], students who ruminate more may use more self-defeating humour, which can result in higher levels of suicide ideation. In a different study, Tucker et al. [38] examined how humour styles moderate the relationship between predictors of suicide ideation and levels of suicide ideation among college students. The predictors they used were thwarted belongingness and perceived burdensomeness, which are known interpersonal predictors of suicide ideation [39]. Tucker et al. [38] found that for students with high levels of affiliative humour, there were weaker relationships between thwarted belongingness and suicide ideation and between perceived burdensomeness and suicide ideation relative to students with low levels of affiliative humour. In contrast, for students with high levels of self-defeating humour there were stronger relationships between these predictors and suicide ideation. Thus, affiliative humour buffered the impact these predictors had on suicide ideation, whereas self-defeating humour exacerbated this relationship. Their study illustrates how positive and negative humour styles may function as risk or protective factors for suicide ideation in a college student sample.

### The present study

The purpose of the present study was to examine associations between humour styles, hopelessness, and suicide ideation, in both student and community samples. As noted, Tucker et al. [38] examined interpersonal predictors of suicide ideation among college students (thwarted belongingness and perceived burdensomeness). Thwarted belongingness and perceived burdensomeness are closely related to social hopelessness: an individual who feels that they do not belong to a social group and perceives themselves as a burden likely experiences hopelessness with respect to their current and future social interactions. Both social and general hopelessness have been found to be positively associated with suicide ideation among college students and adolescents [40–42], although Heisel et al. [41] found that only social hopelessness and depression were able to discriminate between those low and high in suicide ideation. A 13-year longitudinal study found that general hopelessness is one of the best predictors of suicide ideation in community samples, above and beyond both depression and substance abuse [43]. In contrast, for college students, depression is a better predictor for suicide ideation than general hopelessness [44–46]. To build and expand on previous research that has focused on social/interpersonal predictors of suicide ideation in student samples, it is necessary to use both student and non-student samples as well as an intrapersonal measure of hopelessness.

In the present study, two different samples of participants were recruited: a community sample and a student sample. The Beck Hopelessness Scale [47] was used as a measure of general hopelessness (hereafter referred to as “hopelessness”). The two samples allowed for comparisons of the associations between humour styles, hopelessness, and suicide ideation. To our knowledge, no other study has examined the potential moderating effect of humour styles on the relationship between hopelessness and suicide ideation in a community sample. Doing so is especially important, as suicide ideation can affect people of any age and demographic [27]. Our study therefore advances researchers’ understanding of the influence that humour styles have on mental health and the risk factors for suicide ideation.

With respect to moderation effects, we hypothesized that affiliative humour would act to buffer the association between hopelessness and suicide ideation given the proposed social benefits of humour [1]. That is, if one is able to create and maintain healthy social relationships via an affiliative humour style, then these social benefits could partially counteract one’s negative thoughts about the future and thereby reduce the influence of hopelessness on suicide ideation. Similarly, the habitual use of self-enhancing humour to cope with stressful life events and situations could partially counteract one’s negative thoughts about the future and thereby reduce the influence of hopelessness on suicide ideation. In both cases, the humour style (affiliative or self-enhancing) would protect the individual from the detrimental relationship between hopelessness and suicide ideation. On the other hand, a habitual self-defeating humour style could affect one’s social standing as well as their outlook and ability to cope with stressful situations, thereby exacerbating the detrimental relationship between hopelessness and suicide ideation.

Based on the findings of previous research, for the correlational analyses it was hypothesized that 1a) affiliative humour and 1b) self-enhancing humour would be negatively correlated with hopelessness and suicide ideation, whereas 1c) aggressive humour and 1d) self-defeating humour would be positively correlated with hopelessness and suicide ideation. For the regression analyses, it was hypothesized that 2a) hopelessness would be a significant predictor of suicide ideation for both community and student participants while controlling for other variables, 2b) affiliative and 2c) self-enhancing humour would be negatively associated with suicide ideation while controlling for other variables, and 2d) self-defeating humour would be positively associated with suicide ideation while controlling for other variables. With respect to moderation effects, it was hypothesized that 3a) affiliative and 3b) self-enhancing humour would moderate the relationship between hopelessness and suicide ideation, with those with high levels of hopelessness and high levels of affiliative humour or self-enhancing humour experiencing lower levels of suicide ideation than those with low levels of affiliative humour or self-enhancing humour, whereas 3c) self-defeating humour would moderate this relationship differently, with those with high levels of hopelessness and high levels of self-defeating humour experiencing higher levels of suicide ideation than those with low levels of self-defeating humour.

## Method

### Participants

To be eligible for the study, participants had to be at least 18 years of age, currently living in the U.S. or Canada, and able to read and speak English. There were 554 participants recruited for the community sample, with 284 (51.3%) identifying as “male”, 250 (45.1%) as “female”, 7 (1.3%) as “non-binary/third gender”, 1 (0.2%) “prefer not to say”, and 12 (2.2%) not responding. The mean age was 36.66 (*SD* = 9.95), with a range of 18 to 71. Four hundred (72.2%) participants identified as White/European, 55 (9.9%) as African American/Black, 32 (5.8%) as Asian, 23 (4.2%) as Latin American, 11 (2.0%) as Other, 10 (1.8%) as South Asian, 5 (0.9%) as Southeast Asian, 5 (0.9%) as Filipino, 1 (0.2%) as Arab, and 12 (2.2%) did not report.

For the student sample, 208 participants were recruited, with 169 (81.3%) identifying as “female”, 37 (17.8%) identifying as “male”, 1 (0.5%) as “non-binary/third gender”, and 1 (0.5%) not responding. The mean age was 19.86 (*SD* = 2.34), with a range of 18 to 35. Eighty (38.5%) participants identified as White/European, 45 (21.6%) as South Asian, 23 (11.1%) as Asian, 12 (5.8%) as African American/Black, 12 (5.8%) as Filipino, 10 (4.8%) as Latin American, 8 (3.8%) as Other, 6 (2.9%) as Arab, 5 (2.4%) as Southeast Asian, 4 (1.9%) as West Indian, 1 (0.5%) as First Nations, and 2 (1.0%) did not report.

### Measures

#### Demographics questionnaire

Participants completed an author-compiled questionnaire that collected information on age, gender, ethnicity, education level, country of residence, living arrangements, occupation, employment status, annual income range, marital status, and number of children. In addition, they were asked about previous experiences with prescription medication and counselling for depression, as well as diagnoses from mental health professionals.

#### Beck Hopelessness Scale (BHS)

The BHS is a 20-item self-report scale measuring hopelessness using true-false statements [47]. These statements assess an individual’s cognitive schemas involving negative expectancies towards their future and overall pessimism (e.g., “I might as well give up because I can’t make things better for myself”). Psychometric analyses have shown that the scale measures three factors: “feelings about the future”, “loss of motivation”, and “future expectations”. All items include self-based statements (e.g., “I”, “me”, “my”), consistent with the intrapersonal focus of the BHS. Internal consistency (Cronbach’s alpha) was excellent for both the community (α = .91) and student samples (α = .90).

#### Humor Styles Questionnaire (HSQ)

The HSQ is a 32-item self-report questionnaire with four sub-scales measuring four humour styles: affiliative, self-enhancing, aggressive, and self-defeating [17]. Each item is rated on a 7-point Likert scale, ranging from 1 (“totally disagree”) to 7 (“totally agree”). Examples of affiliative, self-enhancing, aggressive, and self-defeating items are “I laugh and joke a lot with my closest friends” (affiliative); “If I am feeling depressed, I can usually cheer myself up with humor” (self-enhancing); “If I don’t like someone, I often use humor or teasing to put them down” (aggressive), and “I will often get carried away in putting myself down if it makes my family or friends laugh” (self-defeating). The items are ordered so the respondent receives one item from each subscale in sequence. There is evidence that the HSQ exhibits no gender bias in the items [48]. Martin et al. [17] reported that the HSQ has good or acceptable internal consistency for all humour style subscales (α = .77-.81), as well as good test-retest reliability (*r* = .80-.85). For the community sample, internal consistency for the affiliative (α = .84), self-enhancing (α = .82), and self-defeating (α = .85) subscales was good, whereas for the aggressive subscale it was acceptable (α = .63). Similarly, for the student sample, internal consistency for the affiliative (α = .82), self-enhancing (α = .81), and self-defeating (α = .86) subscales was good, whereas for the aggressive subscale internal consistency was acceptable (α = .64). Other researchers have reported low internal consistency for the aggressive humour subscale (e.g., .71 in [20]; .70 in [38]), and this should be considered when interpreting any of our results involving this subscale.

#### Hopelessness Depressive Symptom Questionnaire-Suicidality Subscale (HDSQ-SS)

The HDSQ-SS is a four-item self-report sub-scale that measures suicide ideation within the past two weeks [49]. Each item consists of four statements, which are scored from 0–3 (e.g., “I do not have thoughts of killing myself” = 0; “Sometimes I have thoughts of killing myself” = 1; “Most of the time I have thoughts of killing myself” = 2; “I always have thoughts of killing myself” = 3). Tucker et al. [38] reported that the HDSQ-SS has excellent internal consistency (α = .93). Cronbach’s alpha was .93 for the community sample and .92 for the student sample.

#### Procedure

The study received ethics approval from the Conjoint Faculties Research Ethics Board at the University of Calgary prior to data collection (REB20-1979). Student participants were recruited from the University of Calgary using an internet-based research participation system open to students registered in psychology courses. Student participants were provided with bonus credit in a psychology course of their choice for full or partial completion of the study measures (2% added to their final course grade). Students were provided with a brief description of the nature of the study and its measures to allow those uncomfortable with questions about suicidality to decline to participate. Those who consented to participate were connected to the study website (hosted on Qualtrics) via a hypertext link. Participants first provided informed consent and then completed the demographics and the measures described above (BHS, HSQ, HDSQ-SS). For the debriefing, participants were provided with information and contacts on suicide prevention, as well as a suggestion to seek help if they were struggling with negative thoughts. Participants were informed of the potential risks associated with suicidal thoughts, as well as relationships between humour styles and mental health. Participants were able to end their participation at any point and were automatically directed to the debriefing when they elected to discontinue.

Community participants were recruited through the Amazon Mechanical Turk (MTurk) marketplace service. This service allows researchers to crowdsource tasks such as data validation and survey participation via online workers who receive payment [50]. Recent reviews have found that MTurk samples recruited for personality studies [51], addictions research [52], and psychopathology studies [53] are comparable to those collected via traditional methods. A brief pre-screen survey was used to recruit part of the community sample, to increase the likelihood of recruiting participants who had experienced suicide ideation (given that we were uncertain what percentage of prospective MTurk participants would have such thoughts). The pre-screen included brief demographic and mental health questions (age, gender, current and past experiences with depression and anxiety) and one item pertaining to self-harm (“Have you had any experiences with thoughts of self-harm within the past 2 weeks?”). Those who responded “yes” were invited to participate in the study and made up 60.09% of the community sample (note that this item did not inquire about suicide ideation per se, and so affirmative responses were not necessarily indicative of such thoughts). The remaining participants in the community sample were recruited directly to the study via MTurk without using a pre-screen. Note that no pre-screening was used for the student sample, to be consistent with most of the previous studies in this literature [25, 26, 36, 38]. Like student participants, community participants were provided with a brief description of the study and those who consented to participate then followed a link to the study website hosted on Qualtrics. Participants were able to end participation at any point and were automatically directed to the debriefing when they elected to discontinue their participation. Community participants were paid for their participation ($0.20 USD for completing the pre-screen, and $3 USD for partial completion of the measures in the survey, with a bonus $2 USD for completing all the measures in the survey).

#### Data preparation and statistical power analyses

All statistical analyses were carried out using SPSS Statistics (v. 27). Participants who did not complete the majority of the study measures were excluded from all analyses (*n* = 87; 15.7% of community participants, *n* = 13; 6.3% of student participants). Participants who completed the entire survey were asked for their permission to use their data, and their data was excluded from the analyses if they did not provide such permission (*n* = 3; 0.5% of community participants, *n* = 4; 1.9% of student participants). Attention check questions were used to identify participants who were not closely reading the questions. Three attention check questions were embedded in the survey (e.g., “Please select Somewhat Agree”), and participants who did not correctly answer at least 2 of these checks were excluded from the analyses (*n* = 3; 0.5% of community participants, *n* = 1; 0.5% of student participants). Multivariate outliers were identified by calculating Mahalanobis distances. The Mahalanobis distance for each participant was compared to a chi-square distribution, and participants with *p*-values below .001 (*n* = 35, 6.3% of community; *n* = 21, 10.1% of student participants) were excluded from further analyses [54]. The final sample size was 592 participants, consisting of 424 community participants and 168 student participants.

Prior to analyses, normality was checked for independent (BHS) and dependent (HDSQ-SS) variables. In the community sample, both appeared normal, and the BHS had a skewness value of 0.48 and a kurtosis value of −0.73, while the HDSQ-SS had a skewness value of 1.03, and a kurtosis value of −0.14. In the student sample, both appeared normal, and the BHS had a skewness value of 0.94 and a kurtosis value of 0.16, while the HDSQ-SS had a skewness value of 1.45 and a kurtosis value of 1.22. Thus, all checks fell within the normal range. For the moderation analyses, the Process macro for SPSS [55] was used to perform hierarchical regression analyses and follow-up tests of statistically significant moderation effects. All variance inflation factors (VIF) were below 1.26, which indicated that there was no multicollinearity between the predictors and moderators (a VIF greater than 4.0 is generally considered problematic). Statistical power analyses were carried out (using G*Power 3.1 [56]) to determine the power to detect specific increases in R^2^ in the regression analyses due to a moderation effect. For the community sample (*N* = 424), the power to detect an *R*^2^ increase of 2% due to a moderation effect (the smallest statistically significant moderation effect reported by Tucker et al., [38]) was 84%. According to conventions, this would correspond to a “small” effect [57]. The power to detect a 5% increase in R^2^ was 99%. For the student sample (*N* = 168), the power to detect 2% and 5% increases in R^2^ was 45% and 84%, respectively.

## Results

### Demographic characteristics of the samples

Demographic characteristics of community and student participants were compared using a *t*-test and chi-square tests. As expected, community participants were older (*M* = 36.91) than student participants (*M* = 19.83), *t*(514.32) = 33.64, *p* < .001, *d* = 2.01 (correcting for unequal variances). Most of the student participants were women (*n* = 138; 82.6%), whereas the percentage of women in the community sample was much smaller (*n* = 193; 46.1%), *χ*^2^(1) = 64.98, *p* < .001. Community participants differed from student participants in ethnicity, as a larger percentage of community participants were White (*n* = 316; 74.5%, vs. *n* = 70; 41.7%), *χ*^2^(1) = 56.25, *p* < .001. The two samples also differed in their employment status, *χ*^2^(2) = 305.42, *p* < .001. Most of the community participants were employed full-time (*n* = 337; 79.5%), and smaller percentages were employed part-time (*n* = 51; 12.0%) or unemployed (*n* = 33; 7.8%), whereas the majority of the student participants were unemployed (*n* = 86; 51.2%) and few were working full-time (*n* = 3; 1.8%). Community participants differed from student participants in their reported annual income, *χ*^2^(5) = 300.74, *p* < .001. The largest percentage of community participants reported an annual income of $45,000–99,999 (*n* = 176; 41.5%), whereas the largest percentage of student participants reported an annual income of less than $20,000 (*n* = 143; 85.1%).

### Sample comparisons of humour styles and suicide ideation

To further examine how community participants differed from student participants, *t*-tests were used to compare community and student participants on humour styles, predictors of suicide ideation, and suicide ideation. To compensate for multiple comparisons, an alpha of 1% was used for all tests (statistical power to detect a small effect size, *d* = .30, was 76%; for a medium effect size, *d* = .50, power was 99%). Community versus student participant comparisons are shown in Table 1. Community participants had significantly lower scores for affiliative humour than student participants, and significantly higher scores for self-enhancing and aggressive humour. Community participants had significantly higher scores on suicide ideation and hopelessness. In the community sample, 54.8% of participants reported no suicide ideation (a score of 0 on the HDSQ-SS), with a maximum score of 11 (two participants). In the student sample, 65.5% of participants reported no suicide ideation, with a maximum score of 9 (one participant). These comparisons demonstrate differences in both humour usage and general mental well-being between the two samples.

**Table 1:** T-Tests comparing community and student participants on humour styles and psychological measures.

	Community		Student				
Measure	*M*	*SD*	*M*	*SD*	*t*	*p*	*d*
Affiliative humour	37.99	9.15	44.48	6.96	9.30	< .001	0.76
Self-enhancing humour	36.72	8.01	33.65	8.13	4.19	< .001	0.38
Aggressive humour	28.65	7.44	25.64	6.42	4.62	< .001	0.42
Self-defeating humour	32.18	9.90	30.15	9.91	2.25	.025	0.21
Hopelessness	7.04	5.32	5.64	4.61	3.18	.002	0.27
Suicide ideation	1.99	2.70	1.18	1.97	3.99	< .001	0.32

Hopelessness = BHS total, Suicide ideation = HDSQ-SS total, *d* = Cohen’s *d*.

Given that several studies have found differences between men and women in the use of aggressive humour [17, 58–60], *t*-tests were used to compare the humour style scores and psychological measures of men and women in the student and community samples. To compensate for multiple comparisons, an alpha of 1% was used for all tests (statistical power to detect a small effect size, *d* = .30, was 85%; power to detect a medium effect size, *d* = .50, was 99%). These data are shown in Table 2. There were no significant differences in affiliative, self-enhancing, or self-defeating humour, nor in suicide ideation or hopelessness scores. Men had higher aggressive humour scores than women in the community sample, and the effect size (*d* = .32) was similar to the effect size reported by Ruch et al. [60] (*d* = .44). A similar difference was present in the student sample but was not statistically significant. As noted above, however, the low internal consistency of the aggressive humour subscale (< .70) necessitates caution when interpreting any results involving this subscale. Given these results, we did not incorporate gender into the multiple regression analyses reported below.

**Table 2:** T-Tests comparing women and men on humour styles and psychological measures, for community and student participants.

	Women		Men				
	*M*	*SD*	*M*	*SD*	*t*	*p*	*d*
**Measure**	**Community**						
Affiliative humour	38.60	9.00	37.42	9.32	1.31	.192	0.13
Self-enhancing humour	36.47	8.19	37.00	7.79	0.68	.495	0.07
Aggressive humour	27.36	7.85	29.71	6.95	3.22	.001	0.32
Self-defeating humour	31.10	10.29	33.13	9.50	2.10	.037	0.21
Hopelessness	7.15	5.51	6.77	5.07	0.72	.474	0.07
Suicide ideation	2.15	2.83	1.85	2.61	1.12	.265	0.11
	**Student**						
Affiliative humour	44.48	7.06	44.14	6.47	0.24	.811	.05
Self-enhancing humour	33.66	8.10	33.28	8.32	0.23	.818	.05
Aggressive humour	25.16	6.17	27.79	7.32	2.02	.045	.41
Self-defeating humour	30.92	10.20	26.28	7.54	2.32	.022	.47
Hopelessness	5.94	4.70	4.38	3.90	1.67	.096	.34
Suicide ideation	1.29	2.03	0.72	1.60	1.64	.107	.29

Hopelessness = BHS total, Suicide ideation = HDSQ-SS total, *d* = Cohen’s *d*.

### Correlational analyses

Pearson correlations were used to examine associations between variables, for student and community participants separately. To compensate for multiple analyses, an alpha of 1% was used (statistical power to detect a correlation of *r* = ±.30 was 99%). The means, standard deviations, and correlations for community participants are shown in Table 3. As hypothesized (Hypotheses 1a, 1c, and 1d), affiliative humour was negatively correlated with suicide ideation (*r* = −.43), and both aggressive and self-defeating humour were positively correlated with suicide ideation (*r* = .23 and *r* = .37, respectively). These correlations are consistent with those reported by Tucker et al. [38], who used a student sample, although the correlation between aggressive humour and suicide ideation was not statistically significant in their study. As hypothesized (Hypotheses 1a and 1d), affiliative humour was negatively correlated with hopelessness (*r* = −.29), and self-defeating humour was positively correlated with hopelessness (*r* = .24). Contrary to hypotheses (Hypotheses 1b and 1c), self-enhancing humour was negatively correlated with hopelessness (*r* = −.34) but not with suicide ideation (*r* = −.01), and aggressive humour was not positively correlated with hopelessness (*r* = .12).

**Table 3:** Correlations and descriptive statistics for community participants.

Measure	1	2	3	4	5	6
**1.** Affiliative humour	-					
**2.** Self-enhancing humour	.29**	-				
**3.** Aggressive humour	−.18**	.06	-			
**4.** Self-defeating humour	−.32**	.12	.49**	-		
**5.** Hopelessness	−.29**	−.34**	.12	.24**	-	
**6.** Suicide ideation	−.43**	−.01	.23**	.37**	.37**	-
Mean	37.99	36.72	28.65	32.18	7.04	1.99
Standard Deviation	9.15	8.01	7.44	9.90	5.32	2.70

Hopelessness = BHS total, Suicide ideation = HDSQ-SS total.

**p < .001.

Table 4 shows the descriptive statistics and correlations for student participants. Statistical power to detect a correlation of *r* = ±.30 was 92% using an alpha of 1%. As was the case for community participants, and as hypothesized (Hypothesis 1a, 1b, and 1d), affiliative humour and self-enhancing humour were negatively correlated with hopelessness (*r* = −.20 and *r* = −.27, respectively), and self-defeating humour was positively correlated with both hopelessness and suicide ideation (*r* = .45 and *r* = .39, respectively). In contrast to the community sample, and contrary to Hypothesis 1c, aggressive humour was not correlated with suicide ideation (*r* = .12), similar to what was found by Tucker et al. [38] in their student sample. In addition, contrary to Hypothesis 1a, affiliative humour was not negatively correlated with suicide ideation (*r* = .00), which differs from Tucker et al.’s findings.

**Table 4:** Correlations and descriptive statistics for student participants.

Measure	1	2	3	4	5	6
**1.** Affiliative humour	-					
**2.** Self-enhancing humour	.40**	-				
**3.** Aggressive humour	.07	.10	-			
**4.** Self-defeating humour	.11	.01	.20	-		
**5.** Hopelessness	−.20*	−.27**	.13	.45**	-	
**6.** Suicide ideation	.00	−.19	.12	.39**	.54**	-
Mean	44.48	33.65	25.64	30.15	5.64	1.18
Standard Deviation	6.96	8.13	6.42	9.91	4.61	1.97

Hopelessness = BHS total, Suicide ideation = HDSQ-SS total.

*p < .01.

**p < .001.

### Hierarchical regression analyses

To determine whether humour styles significantly moderated the relationship between hopelessness and suicide ideation, interaction terms between hopelessness (BHS scores) and the four different humour styles (affiliative, self-enhancing, aggressive, and self-defeating HSQ scores) were used in hierarchical multiple regression models (using a separate model for each humour style). For each analysis, in Model 1, the centered value of scores on the BHS was entered as a predictor, along with the centered scores of affiliative, self-enhancing, aggressive, or self-defeating humour, with suicide ideation as the dependent variable. In Model 2, the interaction term was entered (BHS x humour style) and tested for significance (change in *R*^2^). Bonferroni corrections were applied to compensate for four analyses, and therefore an alpha of .013 was used for all tests of significance.

### Affiliative humour

The regression analysis results for affiliative humour, for community and student participants, are shown in Table 5. For community participants, Model 1, with hopelessness and affiliative humour as the predictors, was significant, *R*^2^ = .246, *F*(2, 421) = 68.60, *p* < .001, accounting for 24.6% of the variation in suicide ideation. Consistent with hypotheses (Hypotheses 2a and 2b, respectively), both hopelessness (*B* = 0.14, *p* < .001) and affiliative humour (*B* = −0.10, *p* < .001) were significant predictors of suicide ideation. That is, higher levels of hopelessness were associated with higher levels of suicide ideation, while controlling for affiliative humour, and higher levels of affiliative humour were associated with lower levels of suicide ideation, while controlling for hopelessness. Adding the interaction term to the model (Model 2) did not increase *R*^2^ significantly (0.2%), *F*(1, 420) = 0.97, *p* = .326. This outcome indicates that for affiliative humour, there was no significant moderation effect, contrary to Hypothesis 3a. That is, regardless of the level of affiliative humour, higher levels of hopelessness were associated with higher levels of suicide ideation, with affiliative humour providing no buffering effect on the relationship between hopelessness and suicide ideation.

**Table 5:** Results of hierarchical regression analysis testing for moderation of the relationship between hopelessness and suicide ideation by affiliative humour, for community and student participants.

Community Participants	*R* ^2^	Δ*R*^2^	Δ*F*	*B*	*SE*	*t*	*p*
**Model 1**	.246	.246	68.60				< .001
Hopelessness				0.136	0.022	6.04	< .001
Affiliative humour				−0.102	0.013	7.84	< .001
**Model 2**	.248	.002	0.97				.326
Hopelessness				0.140	0.023	6.12	< .001
Affiliative humour				−0.102	0.013	7.76	< .001
Hopelessness x Affiliative humour				−0.002	0.002	0.98	.326
**Student Participants**							
**Model 1**	.300	.300	35.33				< .001
Hopelessness				0.239	0.028	8.41	< .001
Affiliative humour				0.031	0.019	1.64	.102
**Model 2**	.312	.012	2.93				.089
Hopelessness				0.243	0.028	8.56	< .001
Affiliative humour				0.033	0.019	1.77	.078
Hopelessness x Affiliative humour				0.007	0.004	1.71	.089

Hopelessness = BHS total. Affiliative humour = score on the affiliative humour subscale of the HSQ.

For student participants, Model 1, with hopelessness and affiliative humour as predictors, was significant, *R*^2^ = .300, *F*(2, 165) = 35.33, *p* < .001, accounting for 30.0% of the variation in suicide ideation scores. Hopelessness (*B* = 0.24, *p* < .001) was a significant predictor of suicide ideation. As hypothesized (Hypothesis 2a), higher levels of hopelessness were associated with higher levels of suicide ideation, while controlling for affiliative humour. Contrary to hypothesis (Hypothesis 2b), and in contrast to community participants, affiliative humour was not a significant predictor of suicide ideation (*B* = 0.03, *p* = .102). Adding the interaction term (Model 2) did not increase *R*^2^ significantly (1.2%), *F*(1, 164) = 2.93, *p* = .089, indicating that there was no moderation effect. Thus, affiliative humour provided no buffering effect on the relationship between hopelessness and suicide ideation among student participants, contrary to Hypothesis 3a.

### Self-enhancing humour

**Fig 1:**
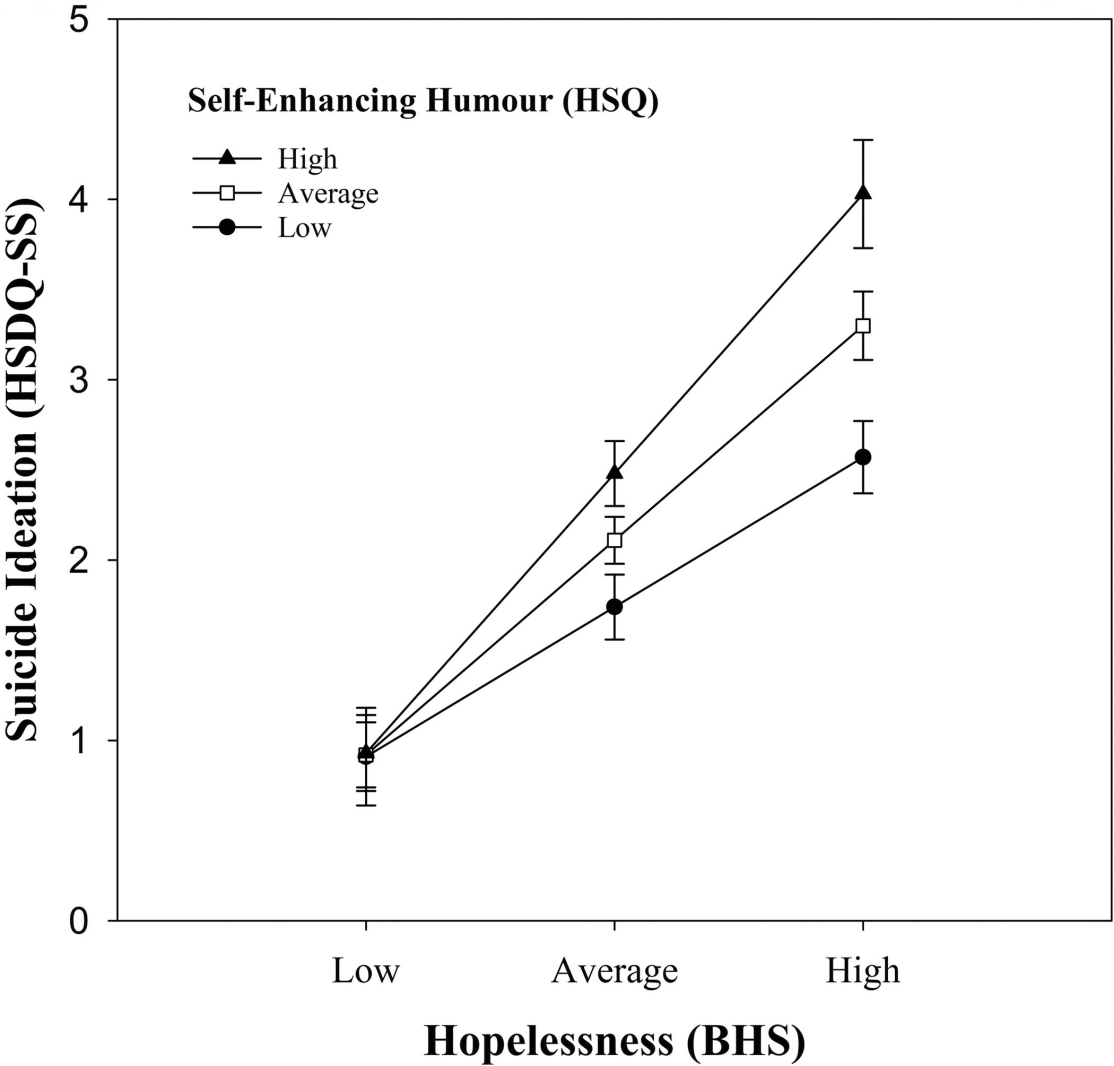
Association between hopelessness and suicide ideation as a function of self-enhancing humour style for community participants. Suicide ideation is the total score on the HSDQ-SS, hopelessness is the total score on the BHS, and self-enhancing humour is the total score of the self-enhancing humour subscale on the HSQ. Error bars represent one standard error of the mean.

The regression analyses for self-enhancing humour, for community and student participants, are shown in Table 6. For community participants, Model 1, with hopelessness and self-enhancing humour as the predictors, was significant, *R*^2^ = .151, *F*(2, 421) = 37.38, *p* < .001, accounting for 15.1% of the variation in suicide ideation. As hypothesized (Hypothesis 2a), hopelessness (*B* = 0.21, *p* < .001) was a significant predictor of suicide ideation. Higher levels of hopelessness were associated with higher levels of suicide ideation, while controlling for self-enhancing humour. Contrary to hypothesis (Hypothesis 2c), self-enhancing humour was positively associated with suicide ideation (*B* = 0.04, *p* = .006), with higher levels of self-enhancing humour associated with higher levels of suicide ideation while controlling for hopelessness. Adding the interaction term to the model (Model 2) increased *R*^2^ by 2.1%, *F*(1, 420) = 10.51, *p* = .001. Interestingly, this moderation effect was in the opposite direction of what was hypothesized (Hypothesis 3b). That is, for community participants with high levels of hopelessness, those with high levels of self-enhancing humour had higher (not lower) levels of suicide ideation than those with low levels of self-enhancing humour (see Fig 1). In fact, the highest levels of suicide ideation were observed among participants with high levels of hopelessness and high levels of self-enhancing humour. Follow-up tests were used to assess the association between self-enhancing humour and suicide ideation at different levels of hopelessness (high, average, and low). These indicated that for community participants with high levels of hopelessness (one SD above the mean; 12.36), there was a positive association between self-enhancing humour and suicide ideation, *t*(420) = 4.23, *p* < .001; higher levels of self-enhancing humour were associated with higher levels of suicide ideation. This was also true when hopelessness levels were average (*M* = 7.04), *t*(420) = 2.89, *p* = .004, but not when hopelessness levels were low (one SD below the mean; 1.72), *t*(420) = 0.06, *p* = .954. Thus, higher levels of self-enhancing humour were associated with higher levels of suicide ideation for community participants with average or high levels of hopelessness.

**Table 6:** Results of hierarchical regression analysis testing for moderation of the relationship between hopelessness and suicide ideation by self-enhancing humour, for community and student participants.

Community Participants	*R* ^2^	Δ*R*^2^	Δ*F*	*B*	*SE*	*t*	*p*
**Model 1**	.151	.151	37.38				< .001
Hopelessness				0.210	0.024	8.64	< .001
Self-enhancing humour				0.044	0.016	2.74	.006
**Model 2**	.172	.021	10.51				.001
Hopelessness				0.223	0.024	9.15	< .001
Self-enhancing humour				0.046	0.016	2.89	.004
Hopelessness x Self-enhancing humour				0.008	0.003	3.24	.001
**Student Participants**							
**Model 1**	.290	.290	33.70				< .001
Hopelessness				0.225	0.029	7.71	< .001
Self-enhancing humour				−0.010	0.017	0.61	.545
**Model 2**	.298	.008	1.89				.171
Hopelessness				0.214	0.030	7.10	< .001
Self-enhancing humour				−0.012	0.016	0.70	.487
Hopelessness x Self-enhancing humour				−0.005	0.004	1.37	.171

Hopelessness = BHS total score. Self-enhancing humour = score on the self-enhancing humour subscale of the HSQ.

For student participants there was a different pattern of results. Model 1, with hopelessness and self-enhancing humour as the predictors, was significant, *R*^2^ = .290, *F*(2, 165) = 33.70, *p* < .001, accounting for 29.0% of the variation in suicide ideation scores. As was the case for community participants, hopelessness (*B* = 0.23, *p* < .001) was a significant predictor of suicide ideation; as hypothesized (Hypothesis 2a), higher levels of hopelessness were associated with higher levels of suicide ideation while controlling for self-enhancing humour. But for student participants, self-enhancing humour was not a significant predictor of suicide ideation (*B* = −0.01, *p* = .545), contrary to Hypothesis 2c. Moreover, there was no moderation effect, as *R*^2^ did not increase significantly when the interaction term (Model 2) was entered (0.8%), *F*(1, 164) = 1.89, *p* = .171. For student participants, self-enhancing humour provided no buffering or exacerbating effect on the relationship between hopelessness and suicide ideation, in contrast to the exacerbating effect observed for community participants.

### Aggressive humour

Table 7 shows the regression analyses results for aggressive humour, for community and student participants. For community participants, Model 1, with hopelessness and aggressive humour as the predictors, was significant, *R*^2^ = .171, *F*(2, 421) = 43.56, *p* < .001, accounting for 17.1% of the variation in suicide ideation scores. Hopelessness (*B* = 0.18, *p* < .001) and aggressive humour (*B* = 0.07, *p* < .001) were significant predictors of suicide ideation. As hypothesized (Hypothesis 2a), higher levels of hopelessness were associated with higher levels of suicide ideation while controlling for aggressive humour, and higher levels of aggressive humour were associated with higher levels of suicide ideation while controlling for hopelessness. Adding the interaction term (Model 2) did not increase *R*^2^ significantly (0.8%), *F*(1, 420) = 4.11, *p* = .043 (using the corrected alpha level of .013). This outcome indicates that aggressive humour was not a significant moderator of the association between hopelessness and suicide ideation.

**Table 7:** Results of hierarchical regression analysis testing for moderation of the relationship between hopelessness and suicide ideation by aggressive humour, for community and student participants.

Community Participants	*R* ^2^	Δ*R*^2^	Δ*F*	*B*	*SE*	*t*	*p*
**Model 1**	.171	.171	43.56				< .001
Hopelessness				0.175	0.023	7.73	< .001
Aggressive humour				0.069	0.016	4.26	< .001
**Model 2**	.179	.008	4.11				.043
Hopelessness				0.181	0.023	7.96	< .001
Aggressive humour				0.073	0.016	4.50	< .001
Hopelessness x Aggressive humour				0.006	0.003	2.03	.043
**Student Participants**							
**Model 1**	.291	.291	33.82				< .001
Hopelessness				0.227	0.028	8.02	< .001
Aggressive humour				0.015	0.020	0.73	.464
**Model 2**	.295	.004	1.00				.319
Hopelessness				0.234	0.029	8.01	< .001
Aggressive humour				0.013	0.020	0.64	.526
Hopelessness x Aggressive humour				−0.005	0.005	1.00	.319

Hopelessness = BHS total score. Aggressive humour = score on the aggressive humour subscale of the HSQ.

For student participants, Model 1, with hopelessness and aggressive humour as the predictors, was significant, *R*^2^ = .291, *F*(2, 165) = 33.82, *p* < .001, accounting for 29.1% of the variation in suicide ideation scores. Hopelessness (*B* = 0.23, *p* < .001) was a significant predictor of suicide ideation. As hypothesized (Hypothesis 2a), higher levels of hopelessness were associated with higher levels of suicide ideation, while controlling for aggressive humour. In contrast to community participants, aggressive humour was not a significant predictor of suicide ideation (*B* = 0.02, *p* = .464). Adding the interaction term (Model 2) did not result in a significant change in *R*^2^ (0.4%), *F*(1, 164) = 1.00, *p* = .319, indicating that there was no moderation effect. Thus, for student participants, aggressive humour was not related to either hopelessness or suicide ideation.

### Self-defeating humour

Table 8 shows the results of the regression analyses for self-defeating humour, for community and student participants. For community participants, Model 1, with hopelessness and self-defeating humour as predictors, was significant, *R*^2^ = .220, *F*(2, 421) = 59.32, *p* < .001, accounting for 22.0% of the variation in suicide ideation scores. Both hopelessness (*B* = 0.15, *p* < .001) and self-defeating humour (*B* = 0.08, *p* < .001) were significant predictors of suicide ideation. As hypothesized, higher levels of hopelessness were associated with higher levels of suicide ideation while controlling for self-defeating humour (Hypothesis 2a), and higher levels of self-defeating humour were associated with higher levels of suicide ideation while controlling for hopelessness (Hypothesis 2d). Adding the interaction term (Model 2) did not increase *R*^2^ significantly (0.7%), *F*(1, 420) = 3.88, *p* = .050 (using the corrected alpha level of .013), indicating that there was no moderation effect. Thus, for community participants, high levels of self-defeating humour did not moderate the relationship between hopelessness and suicide ideation, contrary to Hypothesis 3c.

**Table 8:** Results of hierarchical regression analysis testing for moderation of the relationship between hopelessness and suicide ideation by self-defeating humour, for community and student participants.

Community Participants	*R* ^2^	Δ*R*^2^	Δ*F*	*B*	*SE*	*t*	*p*
**Model 1**	.220	.220	59.32				< .001
Hopelessness				0.150	0.023	6.66	< .001
Self-defeating humour				0.082	0.012	6.74	< .001
**Model 2**	.227	.007	3.88				.050
Hopelessness				0.157	0.023	6.91	< .001
Self-defeating humour				0.088	0.012	7.04	< .001
Hopelessness x Self-defeating humour				0.005	0.002	1.97	.050
**Student Participants**							
**Model 1**	.317	.317	38.21				< .001
Hopelessness				0.193	0.031	6.27	< .001
Self-defeating humour				0.037	0.014	2.61	.010
**Model 2**	.366	.049	12.76				< .001
Hopelessness				0.156	0.032	4.92	< .001
Self-defeating humour				0.046	0.014	3.25	.001
Hopelessness x Self-defeating humour				0.012	0.003	3.57	< .001

Hopelessness = BHS total score. Self-defeating humour = score on the self-defeating humour subscale of the HSQ.

**Fig 2:**
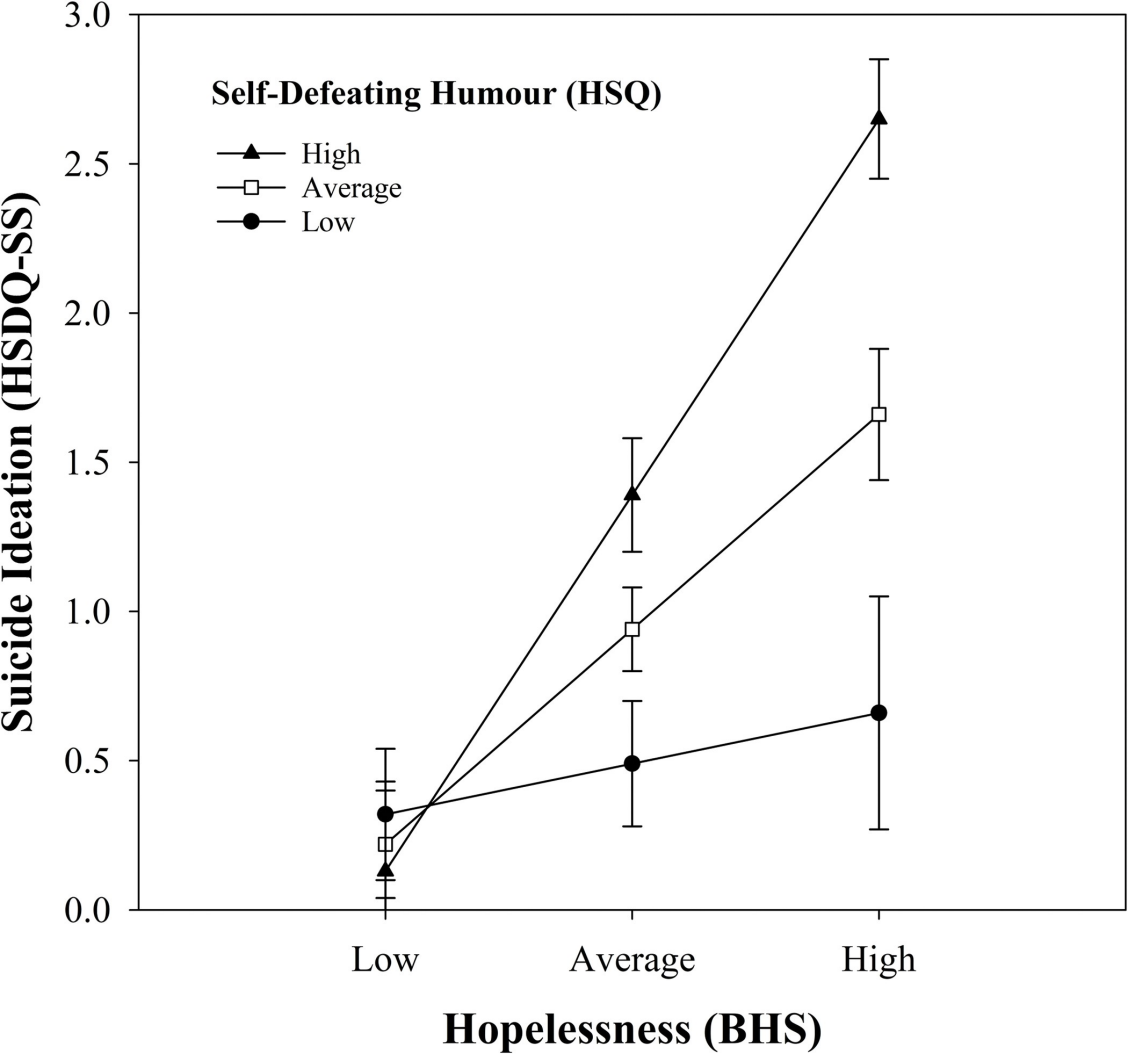
Association between hopelessness and suicide ideation as a function of self-defeating humour style for student participants. Suicide ideation = total score on the HSDQ-SS. Hopelessness = total score on the BHS. Self-defeating humour = total score on the self-defeating humour subscale on the HSQ. Error bars represent one standard error of the mean.

For student participants there was again a different pattern of results. Model 1, with hopelessness and self-defeating humour as the predictors, was significant, *R*^2^ = .317, *F*(2, 165) = 38.21, *p* < .001, accounting for 31.7% of the variation in suicide ideation scores. Both hopelessness (*B* = 0.19, *p* < .001) and self-defeating humour (*B* = 0.04, *p* = .010) were significant predictors of suicide ideation. As was the case for community participants, and as hypothesized, higher levels of hopelessness were associated with higher levels of suicide ideation while controlling for self-defeating humour (Hypothesis 2a), and higher levels of self-defeating humour were associated with higher levels of suicide ideation while controlling for hopelessness (Hypothesis 2d). Unlike the results for community participants, adding the interaction term increased *R*^2^ significantly (4.9%), *F*(1, 164) = 12.76, *p* < .001, reflecting a moderation effect. This moderation effect was in the hypothesized direction (Hypothesis 3c) and is shown in Fig 2. Student participants with high levels of both hopelessness and self-defeating humour had higher levels of suicide ideation than students with high levels of hopelessness and low levels of self-defeating humour. Simple slopes follow-up tests showed that when self-defeating humour was low (one SD below the mean; 20.24), hopelessness scores were not related to suicide ideation, *t*(164) = 0.71, *p* = .481. However, when self-defeating humour was average (*M =* 30.15) and high (one SD above the mean; 40.06), there was a positive relationship between hopelessness and suicide ideation, *t*(164) = 4.92, *p* < .001; *t*(164) = 7.33, *p* < .001, respectively. That is, the strength of the association between hopelessness and suicide ideation was moderated by self-defeating humour. For student participants, self-defeating humour appears to be a maladaptive humour style that can exacerbate the association between hopelessness and suicide ideation. For community participants this moderation effect was not statistically significant, although it should be noted that the trend in the data was identical, which could indicate that the effect is present but not as strong.

## Discussion

The purpose of this study was to determine whether humour styles moderate the relationship between hopelessness and suicide ideation. Previous research addressing this question has primarily used college student samples [25, 26, 36] and interpersonal predictors of suicide ideation [38], which has limited the generalizability of findings. The present study used an intrapersonal measure of hopelessness to assess the association between humour styles and suicide ideation in both student and community samples. Our findings inform researchers and mental health professionals as to the beneficial and detrimental associations between humour styles and mental health.

There were several hypotheses tested in this study and the results both converged with and diverged from these hypotheses. Hypotheses 1a and 1b, that affiliative and self-enhancing humour would be negatively correlated with hopelessness and suicide ideation, were partially supported. For community participants, both affiliative and self-enhancing humour were negatively correlated with hopelessness, whereas only affiliative humour was negatively correlated with suicide ideation. For student participants, both affiliative and self-enhancing humour were negatively correlated with hopelessness, but neither was correlated with suicide ideation. The differences in the strength of the associations between these adaptive humour styles and hopelessness and suicide ideation point to important differences between student and community participants.

Hypotheses 1c and 1d, that aggressive and self-defeating humour would be positively correlated with hopelessness and suicide ideation, were also partially supported. For community participants, only self-defeating humour was positively correlated with hopelessness, while both aggressive and self-defeating humour were positively correlated with suicide ideation. For student participants, only self-defeating humour was positively correlated with both hopelessness and suicide ideation. These correlations between maladaptive humour styles and suicide ideation replicate those observed by Tucker et al. [38] in their student sample. In addition, aggressive humour did not significantly correlate with suicide ideation, whereas self-defeating humour positively correlated with suicide ideation, which was also the case in Tucker et al.’s study.

With respect to the regression analyses and predictions, Hypothesis 2a, that hopelessness would be an independent predictor of suicide ideation, was fully supported. Hopelessness was a significant predictor of suicide ideation in every analysis, accounting for variation in suicide ideation beyond that accounted for by humour style. Hypotheses 2b and 2c, that affiliative and self-enhancing humour styles would be negatively related to suicide ideation, were partially supported. This was true for affiliative humour in the community sample, but neither affiliative nor self-enhancing humour were significant predictors in the student sample. Moreover, contrary to prediction, self-enhancing humour was positively associated with suicide ideation in the community sample, which implies that high levels of self-enhancing humour may be maladaptive for community participants. Hypothesis 2d, that self-defeating humour would be a positive predictor of suicide ideation, was fully supported. In both community and student samples, higher levels of self-defeating humour predicted suicide ideation while controlling for hopelessness. Together these findings show that there were inconsistent relationships between humour styles, hopelessness, and suicide ideation in community and student participants.

With respect to the moderation analyses, Hypotheses 3a and 3b, that affiliative and self-enhancing humour styles would moderate the relationship between hopelessness and suicide ideation (reducing the level of suicide ideation), was not supported. Affiliative humour was not a significant moderator in either sample, and for community participants, self-enhancing humour had an exacerbating effect on the relationship between hopelessness and suicide ideation. That is, community participants with high levels of self-enhancing humour and high levels of hopelessness tended to have the highest levels of suicide ideation. Conversely, participants with low levels of self-enhancing humour generally had lower levels of suicide ideation regardless of their level of hopelessness. This outcome again suggests that high levels of self-enhancing humour can be maladaptive for community participants. This is in contrast to previous studies with student samples that found high levels of self-enhancing humour to be beneficial to various mental health measures [17, 25]. On the other hand, Tucker et al. [26] did not find self-enhancing humour to significantly moderate the relationship between SAD and MDD, and Tucker et al. [38] found no moderation effect for self-enhancing humour in the relationship between interpersonal predictors (thwarted belongingness and perceived burdensomeness) and suicide ideation. These inconsistent findings suggest that high levels of self-enhancing humour may not be as beneficial as previously thought, particularly for more severe mental health outcomes such as MDD and suicide ideation.

Why would high levels of self-enhancing humour exacerbate the association between hopelessness and suicide ideation? One explanation is that habitual use of self-enhancing humour may be less effective in very distressing situations, such as when one is experiencing a great deal of hopelessness. In a study that examined how coping humour relates to depression, loneliness, and self-esteem, Overholser [61] found that for those who scored low in coping humour (as measured by the Coping Humor Scale [8]), scores of humor creativity were negatively correlated with depression and loneliness and positively correlated with self-esteem. This was not true for those high in coping humour, showing that these relationships were specific to individuals who use coping humour infrequently. Overholser suggested that habitually using humour to cope may render it ineffective in certain situations. Given that self-enhancing humour is closely related to coping humour [17], it may be the case that habitual use of self-enhancing humour could elicit the same counterintuitive effect. A related possibility is that those who habitually use self-enhancing humour to deal with minor unpleasant events and feelings may find it distressing when this humour style is not an effective coping strategy in more difficult situations, such as when experiencing strong feelings of hopelessness. If so, this may amplify their feelings of hopelessness and ultimately lead to more suicide ideation. That is, a self-enhancing humour style may not be effective for overcoming such negative thoughts, and those who rely on it habitually are not effectively protected when experiencing these cognitions.

The final hypothesis (Hypothesis 3c), that self-defeating humour would exacerbate the relationship between hopelessness and suicide ideation, was only supported in the student sample. Students with high levels of hopelessness and high levels of self-defeating humour reported higher levels of suicide ideation. This outcome replicated similar findings in previous studies that used college student samples [26, 38]. We noted that although this moderation effect was not statistically significant (*p* = .050) in the community sample (despite the larger sample size), the trend in the data was identical, which could indicate that these associations are present but weaker. Our results confirm that self-defeating humour is a maladaptive form of humour, and that its habitual use can exacerbate the relationship between predictors of suicide ideation and the experience of suicide ideation in student samples. Establishing whether this is also true in community samples requires further research.

Why would high levels of self-defeating humour exacerbate the association between hopelessness and suicide ideation? Self-defeating humour has been found to be positively correlated with loneliness and shyness [62, 63], and negatively correlated with social competence [64], which suggests that this humour style is linked to poor social interactions, a known risk factor for mental health [65, 66]. Those who habitually use self-defeating humor may have fewer beneficial social interactions and relationships (as this humour style is generally not viewed as a positive attribute by others), and poor social relationships can magnify feelings of loneliness, social isolation, and feelings of hopelessness, as one may lose hope in their ability and prospects for improving such relationships. The fact that participants in the student sample appeared to be more susceptible to the detrimental association between self-defeating humour and suicide ideation is possibly related to the greater influence that peer relationships have for younger adults [67–69].

Finally, the demographics analyses revealed that there were a few notable differences between the humour styles of men and women, with men in the community sample using more aggressive humour (as reported in previous studies; [17, 58–60]). There were also differences between the community and student samples, with the community participants using less affiliative humour, and more self-enhancing and aggressive humour. Taken together, our findings suggest that the demographics of the samples used in studies examining the relationships between humour styles and mental health are an important consideration.

### Limitations and directions for future research

There are several limitations that should be considered when interpreting the findings of this study. The first is the issue of causality. For studies examining associations between humour styles and mental health a significant limitation is the inability to make causal claims, as correlational studies do not allow one to determine whether a given humour style preceded the mental health challenge or vice versa [17]. On the one hand, it seems intuitive that if an individual uses a maladaptive humour style habitually this could lead to a more negative outlook on life (hopelessness) and detrimental effects on their social interactions and standing (thwarted belongingness, perceived burdensomeness). On the other hand, an individual who develops a negative outlook on their life or social standing may be more inclined to adopt a maladaptive humour style (one that is not conducive to coping or positive social interactions). Ultimately, it is impossible to make definitive claims about causation in any correlational study given that there is no way to delineate the sequence of causal events. Researchers should use the findings from this study and others to inform the design of longitudinal studies better suited to making causal claims. If, for example, a large group of individuals’ humour styles and mental health characteristics were measured regularly over time, then one could determine whether a specific humour style preceded a mental health challenge.

With respect to the generalizability of our study’s findings, we note that a recent study comparing Eastern to Western cultures reported that Eastern cultures are less likely to report using humour to cope [70]. Given that the samples in our study were recruited from North America, this would suggest that cross-cultural studies will be necessary to establish the generalizability of our findings. In addition, while several studies have found that Mturk samples are comparable to those recruited by traditional means, Arditte et al. [71] noted that their results indicated that MTurk workers were more likely to report clinical symptoms associated with social anxiety and depression than traditional community or epidemiological samples. If this was true for our sample, it is not clear what impact this may have had on our results. It is also unclear what influence the COVID-19 pandemic may have had on our study, as the data were collected amidst the pandemic, and there is some evidence that rates of suicide ideation increased globally due to the pandemic [72]; although see [73]. Finally, although aggressive humour was not found to moderate any associations in our study, the internal consistency of the aggressive humour subscale of the HSQ was not high (.63 in the community sample and .64 in the student sample) and therefore the differences we observed for this humour style (as well as the absence of differences) should be interpreted with caution.

Our most important recommendation for future research is to focus on recruiting diverse samples of participants when examining the relationships between humour styles and mental health. Previous research has relied on college student samples that typically include a large percentage of young women [25, 26, 36, 38]. Considering the many differences found in this study between community and student participants and between men and women, and given the possibility of cultural differences in the use of humour to cope [70], a focus on recruiting non-Western, non-student samples will be necessary to reach a better understanding of the beneficial and detrimental associations between humour styles and mental health.

### Implications

The results of this study provide further evidence that a self-defeating humour style may be especially harmful for those most vulnerable to serious mental health outcomes such as suicide ideation, and they also suggest that self-enhancing humour may be similarly maladaptive. One of the implications of these findings is that the HSQ could be used as a tool to help identify those at higher risk for suicide ideation. That is, if the HSQ can identify individuals using high levels of self-defeating or self-enhancing humour, this information, combined with standard mental health assessments, could be used to identify those who are at a higher risk for suicide ideation and suicide attempts. Vulnerable individuals could then be educated on these relationships and their potential harm, and this awareness could help individuals modify their humour style so that it is more conducive to coping with adversities and facilitating healthy social relationships.

Our findings also contribute to future research on the effectiveness of humour training programs. There are several studies showing the potential for training vulnerable populations to use humour to ease their mental health symptoms [74–76]. A better understanding of how humour styles influence various mental health relationships can inform the design of such humour training programs. For example, individuals experiencing high levels of hopelessness can learn to reduce their use of maladaptive humour and thereby potentially reduce the risk of experiencing high levels of suicide ideation. There is evidence that people who participate in such programs enjoy them and rate them positively [75] and would seek out additional training if available [76]. Our results suggest that self-defeating and self-enhancing humour would be especially useful to target depending on an individual’s demographic. Humour training programs have the potential to contribute to openly available mental health programs that aim to prevent detrimental mental health relationships from developing.

## Conclusion

While there have been many studies that have examined associations between humour and mental health, only a few of these studies have focused on suicide ideation and most have done so using college student samples. Our study confirms that humour styles are related to one of the most adverse mental health outcomes (suicide ideation) and points to important diversity in the nature of those relations. Notably, we found an association between self-enhancing humour and suicide ideation only for community participants, and an association between self-defeating humour and suicide ideation only for student participants. Our results suggest that future studies should focus on recruiting diverse samples to move this research forward. Like other researchers, we conclude that education targeted to specific humour styles can inform the development of humour training programs designed to benefit mental health, and that consideration of humour styles in mental health assessments could assist in identifying those at higher risk for suicide ideation.
